# Associations of Renalase With Blood Pressure and Hypertension in Chinese Adults

**DOI:** 10.3389/fcvm.2022.800427

**Published:** 2022-02-24

**Authors:** Yang Wang, Chen Chen, Gui-Lin Hu, Chao Chu, Xiao-Yu Zhang, Ming-Fei Du, Ting Zou, Qing Zhou, Yue-Yuan Liao, Qiong Ma, Ke-Ke Wang, Yue Sun, Dan Wang, Yu Yan, Yan Li, Hao Jia, Ze-Jiaxin Niu, Xi Zhang, Lan Wang, Zi-Yue Man, Wei-Hua Gao, Chun-Hua Li, Jie Zhang, Ke Gao, Hui-Xian Li, John Chang, Gary V. Desir, Wan-Hong Lu, Jian-Jun Mu

**Affiliations:** ^1^Department of Cardiovascular Medicine, First Affiliated Hospital of Xi'an Jiaotong University, Xi'an, China; ^2^Key Laboratory of Molecular Cardiology of Shaanxi Province, Xi'an, China; ^3^Department of Cardiology, Northwest Women's and Children's Hospital of Xi'an Jiaotong University Health Science Center, Xi'an, China; ^4^National Engineering Research Center for Beijing Biochip Technology, Beijing, China; ^5^Department of Nephrology, First Affiliated Hospital of Xi'an Jiaotong University, Xi'an, China; ^6^Department of Cardiology, Xi'an International Medical Center Hospital, Xi'an, China; ^7^Department of Cardiology, Xi'an No.1 Hospital, Xi'an, China; ^8^Department of Ophthalmology, Xi'an People's Hospital, Xi'an, China; ^9^Department of Cardiology, Xi'an People's Hospital, Xi'an, China; ^10^Department of Medicine, Yale University School of Medicine, New Haven, CT, United States; ^11^Department of Medicine, Veterans Administration Healthcare System, West Haven, CT, United States

**Keywords:** renalase, hypertension, gene polymorphism, blood pressure, renal puncture biopsy

## Abstract

**Objective:**

Renalase, a novel secretory flavoprotein with amine oxidase activity, is secreted into the blood by the kidneys and is hypothesized to participate in blood pressure (BP) regulation. We investigated the associations of renalase with BP and the risk of hypertension by examining renalase single nucleopeptide polymorphism (SNPs), serum renalase levels, and renal expression of renalase in humans.

**Methods:**

① Subjects (*n* = 514) from the original Baoji Salt-Sensitive Study cohort were genotyped to investigate the association of renalase SNPs with longitudinal BP changes and the risk of hypertension during 14 years of follow-up. ② Two thousand three hundred and ninety two participants from the Hanzhong Adolescent Hypertension Study cohort were used to examine the association of serum renalase levels with hypertension. Renalase expression in renal biopsy specimens from 193 patients were measured by immunohistochemistry. ③ Renalase expression was compared in hypertensive vs. normotensive patients.

**Results:**

① SNP rs7922058 was associated with 14-year change in systolic BP, and rs10887800, rs796945, rs1935582, rs2296545, and rs2576178 were significantly associated with 14-year change in diastolic BP while rs1935582 and rs2576178 were associated with mean arterial pressure change over 14 years. In addition, SNPs rs796945, rs1935582, and rs2576178 were significantly associated with hypertension incidence. Gene-based analysis found that renalase gene was significantly associated with hypertension incidence over 14-year follow-up after adjustment for multiple measurements. ② Hypertensive subjects had higher serum renalase levels than normotensive subjects (27.2 ± 0.4 vs. 25.1 ± 0.2 μg/mL). Serum renalase levels and BPs showed a linear correlation. In addition, serum renalase was significantly associated with the risk of hypertension [*OR* = 1.018 (1.006–1.030)]. ③ The expression of renalase in human renal biopsy specimens significantly decreased in hypertensive patients compared to non-hypertensive patients (0.030 ± 0.001 vs. 0.038 ± 0.004).

**Conclusions:**

These findings indicate that renalase may play an important role in BP progression and development of hypertension.

## Introduction

Hypertension is one of the most common diseases, and is also a major risk factor for cardiovascular and cerebrovascular diseases and chronic kidney disease (CKD) (1). Several studies have shown that sympathetic over-activity is crucial in the pathogenesis of hypertension, and interventions of sympathetic deactivation have been shown to lower BP levels (2). Therefore, identifying novel mechanisms of sympathetic regulation would enhance our understanding of biological mechanisms of blood pressure (BP) regulation, and might facilitate the development of specific targeted drugs for hypertension.

Renalase, firstly discovered in 2005, is a 342-amino-acid flavoprotein with monoamine oxidase activity. It is highly expressed in the kidneys and heart, and circulates in the blood to modulate cardiac function and BP (3, 4). In animal studies, renalase deficiency has been shown to increase BP (5), and recombinant renalase has a hypotensive effect (6, 7). In humans, serum renalase levels are higher in hypertensive than normotensive individuals (8, 9). By contrast, Schlaich et al. (10) found that serum renalase was higher in normotensive controls than in patients with resistant hypertension. In addition, it had been showed that plasma renalase was not different between hypertensive and nomotensive groups (11). Previous human and clinical studies examined small cohorts, and yielded conflicting (8–14). Therefore, the relationships of circulating renalase with BP levels and hypertension in the general population are still unclear. In addition, the correlation between serum and kidney renalase in hypertensive patients needs to be further clarified.

Human renalase (gene name: *RNLS*) is encoded by a 311 Kbp gene with 10 exons located on chromosome 10q23.33 (4). An association between *RNLS* gene and hypertension was demonstrated in several studies. Zhao et al. (15) discovered for the first time in the Han Chinese population that the renalase encoding gene was a new susceptibility gene for hypertension, and its genetic variations might affect BP. *RNLS* polymorphisms have also reported to be associated with hypertension in hemodialysis patients and type 2 diabetes patients (16, 17). However, some studies failed to replicate this association (18, 19). Most importantly, all previous studies were cross-sectional. Whether genetic variations in *RNLS* gene can predict the hypertension incidence over time has not been investigated.

Therefore, in our study, we used both single marker-based and gene-based analyses to examine the relationships of *RNLS* gene with longitudinal BP changes and incident hypertension in a family-based cohort. In addition, we also used our previously established cohort to assess the association between serum renalase levels and the risk of hypertension. Lastly, we compared kidney renalase expression levels in persons with and without hypertension.

## Methods

The entire study was divided into three sections: a longitudinal cohort study, a cross-sectional cohort study and a case-control study.

### Section 1: A Longitudinal Cohort Study to Examine the Associations of *RNLS* Gene With Longitudinal BP Changes and Hypertension Incidence

From April to November 2004, we established the cohort of Baoji Salt-Sensitive Study, which recruited 514 adults from 124 families in seven villages in Baoji city, Shaanxi, China. The detailed study design has been published previously (20–22). To identify the associations of potential genetic variants with longitudinal BP changes and the incidence of hypertension, we followed this cohort in 2009, 2012, and 2018. During each follow-up, the same 3-day examination as that of the baseline period was performed. Data on the development of hypertension and use of antihypertensive drugs was obtained by use of a standard questionnaire. During each of the three 3-day follow-up visits, three BP measurements were obtained. The mean of the nine BP measurements was calculated for the current analysis.

### Section 2: A Cross-Sectional Cohort Study to Study the Association of Serum Renalase Levels With the Risk of Hypertension

The study population was derived from the Hanzhong Adolescent Hypertension Study. Established in 1987, this ongoing prospective, population-based cohort study of 4,623 adolescents underwent serial follow-ups to investigate the development of cardiovascular risk factors originating from children and young adults. Details of the study protocol have been published elsewhere (23–25). To explore the association of circulating renalase with the risk of hypertension, we used cross-sectional analysis of the most recent follow-up data in 2017. The selection process of the subjects is described in Supplementary Figure 1. A total of 2,780 subjects were followed up in 2017. Participants were excluded if they had missing BP levels (*n* = 28), laboratory data on serum or urine (*n* = 352), height and weight (*n* = 1), or if they had a self-identified history of cardiovascular disease, stroke or renal failure (*n* = 7), leaving 2,392 participants for the current analysis.

### Section 3: A Case-Control Study to Examine the Renalase Expressions in Human Renal Tissue of Hypertensive Patients

Patients (*n* = 193) who had undergone renal biopsy for work-up of proteinuria, chronic kidney disease, and acute kidney injury were recruited from December 2018 to November 2019 in the Renal Division of First Affiliated Hospital of Xi'an Jiaotong University. Clinical and anthropometric data were extracted for analysis. The exclusion criteria were as follows: (1) history of infectious diseases, chronic heart disease, stroke, chronic obstructive pulmonary disease and abnormal liver function; (2) estimated glomerular filtration rate (eGFR) <90 mL/min/1.73 m^2^ on admission and (3) inability or unwillingness to participate in the study. The renal biopsy diagnoses included membranous nephropathy (*n* = 83), IgA nephropathy (*n* = 35), mesangial and proliferative nephritis (*n* = 34), systemic lupus erythematosus (*n* = 20), purpura nephritis (*n* = 9), focal segmental glomerulosclerosis (*n* = 4), renal amyloidosis (*n* = 3), diabetic nephropathy (*n* = 3), vasculitis (*n* = 1), and proliferative endocapillary glomerulonephritis (*n* = 1).

The Ethics Committee of the First Affiliated Hospital of Xi'an Jiaotong University approved the study protocol (code: 2015–128). Each participant provided written informed consent. This study follows the principles of the Declaration of Helsinki, and all studies procedures were carried out in accordance with institutional guidelines (ClinicalTrials.gov, registration number: NCT02734472).

### BP Measurement and Definitions

BP was measured by the trained staff using a standard mercury sphygmomanometer as previously described (24, 26, 27). The mean arterial pressure (MAP) was calculated as DBP + [1/3 × (SBP–DBP)]. Hypertension was defined as SBP of ≥140 mm Hg, DBP ≥ 90 mm Hg or as the use of antihypertensive medications. High-normal blood pressure was SBP 120–139 and/or DBP 80–89. Grade 1 hypertension was SBP 140–159 and/or DBP 90–99. Grade 2 hypertension was SBP ≥ 160 and/or DBP ≥ 100 as recommended by 2020 International Society of Hypertension (ISH) hypertension practice guidelines (28). The subtypes of hypertension were further defined as isolated systolic hypertension (ISH: SBP ≥ 140 mmHg and DBP <90 mmHg), isolated diastolic hypertension (IDH: SBP <140 mmHg and DBP ≥ 90 mmHg), and systolic diastolic hypertension (SDH: SBP ≥ 140 mmHg and DBP ≥ 90 mmHg) in the absence of antihypertensive treatment (28).

### Blood Biochemical Analyses

Triglycerides, total cholesterol, high-density lipoprotein (HDL), low-density lipoprotein (LDL), aspartate aminotransferase (AST), alanine aminotransferase (ALT), serum uric acid, serum Na^+^ and K^+^, serum phosphate, serum creatinine, blood urea nitrogen, fasting glucose levels, C-reactive protein, glycosylated hemoglobin and urinary albumin-creatinine ratio (uACR) were assessed by an automatic biochemical analyzer (Hitachi, Tokyo, Japan). Details of these assays were described previously (24, 26, 27). Serum renalase levels were measured by using commercially available enzyme-linked immunosorbent assay (ELISA) kits (Cloud-Clone Corp., Houston, TX, USA).

### Immunohistochemistry Staining

Immunohistochemical (IHC) staining was performed as previously reported (29–31). Briefly, serial 5 μm paraffin-embedded fixed tissue sections were dewaxed and rehydrated. These sections were incubated with renalase antibody (Product code: ab178700, Abcam, USA 1:200) at 4°C overnight, and with secondary antibody at room temperature for 30 min. The sections were photographed by an upright fluorescence microscopic imaging system (BX51 Olympus, Japan). To evaluate the expression of renalase, 10 non-overlapping fields (magnification × 40) were randomly selected from each kidney section. The immunoperoxidase stain was quantified using the Image Pro Plus 6.0 imaging software (Media Cybernetics, USA). Data were expressed as the mean optical density in each field area. Details of this methods were described before (29, 30).

### SNP Selection and Genotyping

Ten tagging SNPs in *RNLS*: rs10509540, rs7922058, rs999951, rs10887800, rs796945, rs1935582, rs7076491, rs2296545, rs2576178 and rs17109290, were selected from *RNLS* gene using the National Center for Biotechnology Information database and the Genome Variation Server database and based on the following criteria: tag SNPs in the CHB and Asian database selected by the Haploview 4.2 software (Broad Institute, Cambridge, MA, USA) with Hardy-Weinberg equilibrium (HWE) *P* ≥ 0.05, a minor allele frequency (MAF) ≥ 0.05, and *r*^2^ ≥ 0.8. All the genotyping experiments were done by CapitalBio (CapitalBio Corp, Beijing, China) as described previously (20, 32, 33).

### Statistical Analyses

For the analyses in family-based cohort study, quality control, including genotyping call rate, Mendelian consistency, minor allele frequency and Hardy-Weinberg equilibrium on parental SNP data, was conducted by PLINK software (version 1.9). Associations of each SNP with longitudinal BP changes were assessed by mixed-effect linear regression models in three genetic models (dominant, recessive, and additive) (32–34). Regarding the analyses of hypertension incidence, 51 participants with hypertension at baseline were excluded. We examined the additive association between each SNP and incident hypertension by using a generalized linear mixed model, which permits multilevel modeling when the response variable follows a binary distribution (e.g., incident hypertension). All models were adjusted for the fixed effects of age, sex, and BMI, and the random effect for familial correlations using *glmer* function in *lme4* R package.

Gene-based analysis was also performed to evaluate the overall association of a candidate gene with BP changes over time and incident hypertension (32, 34, 35). The truncated product method (TPM), which combines *P* values from single SNP association analyses, is an approach that would evaluate the association between a candidate gene and a trait. Gene-based analysis was performed using *R* software (version 3.0.1; http://www.r-project.org).

For the analyses in the cross-sectional cohort study, we performed linear and logistic regression analyses to test associations of serum renalase with BP levels and the risk of hypertension. These analyses were multivariate, adjusting for traditional cardiovascular risk factors and potential confounders. In the observational study, Student's *t*-test was used to determine statistical differences between two groups. All statistical analyses were conducted using SPSS 16.0 (SPSS, Inc., Chicago, IL). *P* < 0.05 was considered statistically significant.

## Results

### Analyses for Associations of Renalase SNPs With Longitudinal BP Changes and Hypertension Incidence in the Longitudinal Cohort Study

Participants were 49 years old and had had a BMI of 22.2 kg/m^2^. Their mean baseline SBP, DBP, and MAP were 115.2, 71.3, and 86.0 mmHg, respectively. During 14 years of follow-up, average SBP, DBP, and MAP increased by 21.2, 7.9, and 12.3 mmHg, respectively, and 160 (53.9%) subjects who were normotensive at baseline developed hypertension (Table 1).

**Table 1:** Characteristics of the study participants at baseline and during follow-ups.

**Characteristics**	**Baseline in 2004**	**Follow-up in 2009**	**Follow-up in 2012**	**Follow-up in 2018**
Gender (M/F)	267/247	208/204	185/171	155/142
Age (years)	48.6 ± 0.9	53.3 ± 0.7	56.6 ± 0.2	62.3 ± 1.4
Body mass index (kg/m^2^)	22.2 ± 0.2	22.4 ± 0.2	23.6 ± 1.0	24.6 ± 0.2
SBP (mmHg)	115.2 ± 0.8	120.0 ± 0.9	129.6 ± 1.0	136.4 ± 1.0
DBP (mmHg)	71.3 ± 0.4	75.8 ± 0.5	77.9 ± 0.6	79.2 ± 0.7
MAP (mmHg)	86.0 ± 0.5	90.5 ± 0.6	95.1 ± 0.6	98.3 ± 0.7
Fasting glucose (mg/dl)	86.9 (80.9–94.4)	91.5 (86.0–99.1)	92.6 (86.7–100.8)	90.8 (84.8–97.5)
Total cholesterol (mg/dl)	155.5 (138.5–177.6)	157.7 ± 29.0	162.4 (145.7–186.4)	178.7 ± 35.3
Triglycerides (mg/dl)	112.7 (82.9–158.5)	129.3 (94.5–175.5)	119.0 (87.0–167.4)	126.5 (91.6–183.7)
HDL (mg/dl)	47.4 ± 11.2	50.9 ± 11.6	49.9 (42.7–58.6)	50.0 (43.2–61.6)
Hypertension at baseline (*n*, %)	51 (9.9)	–	–	–
Hypertension incidence (*n*, %)^*^	–	77 (18.9)	103 (28.9)	160 (53.9)

*SBP, systolic blood pressure; DBP, diastolic blood pressure; MAP, mean arterial pressure; HDL, high-density lipoprotein*.

* *Participants with hypertension at baseline were excluded*.

*Non-normally distributed variables are expressed as the median (interquartile range). All other values are expressed as mean ± SE or n, %*.

The genomic location, minor allele frequency, and Hardy-Weinberg test for each SNP are shown in Supplementary Table 1. No SNPs deviated statistically significantly from Hardy-Weinberg equilibrium.

Table 2 shows the associations of each SNP in *RNLS* gene with 5-year change (2004–2009), 8-year change (2004–2012), and 14-year change (2004–2018) in BP levels. SNPs rs10509540, rs999951, and rs7076491 were significantly associated with 5-year change in SBP while SNP rs1935582 was associated with 5-year change in DBP. SNP rs7922058 was associated with 14-year change in SBP, and rs10887800, rs796945, rs1935582, rs2296545, and rs2576178 were significantly associated with 14-year change in DBP while rs1935582 and rs2576178 were associated with MAP change over 14 years after Bonferroni correction. In addition, gene-based analyses found that *RNLS* was significantly associated with longitudinal DBP change (*P*_TPM_ = 1.48e^−5^) and MAP change (*P*_TPM_ = 0.0085) over a 14-year follow-up (Table 3).

**Table 2:** Association of *RNLS* SNPs with blood pressure changes from baseline to the follow-ups.

**SNP**	**BP (2004–2009)**			**BP (2004–2012)**			**BP (2004–2018)**		
	**SBP change**	**DBP change**	**MAP change**	**SBP change**	**DBP change**	**MAP change**	**SBP change**	**DBP change**	**MAP change**
rs10509540	**0.035** *^***a***^*	0.521	0.809	0.434	0.459	0.946	0.274	0.902	0.590
rs7922058	0.528	0.438	0.933	0.986	0.148	0.396	**0.038** *^***a***^*	0.626	0.966
rs999951	**0.035** *^***a***^*	0.346	0.960	0.763	0.259	0.591	0.424	0.678	0.827
rs10887800	0.593	0.289	0.705	0.758	0.297	0.696	0.329	**0.027**	0.077
rs796945	0.942	0.085	0.295	0.952	0.467	0.732	0.290	**0.044**	0.095
rs1935582	0.613	**0.006**	0.051	0.932	0.149	0.398	0.451	**0.001**	**0.019**
rs7076491	**0.049** *^***a***^*	0.732	0.754	0.897	0.367	0.657	0.784	0.658	0.906
rs2296545	0.260	0.295	0.250	0.810	0.220	0.407	0.388	**0.031**	0.087
rs2576178	0.490	0.564	0.575	0.919	0.303	0.577	0.561	**0.028** *^***a***^*	**0.039** *^***a***^*
rs17109290	0.217	0.931	0.621	0.665	0.650	0.992	0.357	0.459	0.356

*All models were adjusted for the fixed effects of age, sex, and BMI, and the random effect for familial correlations. For associations that were not significant under any model, P values for an additive model are listed. All genetic models are based on the minor allele of each SNP*.

*BP, blood pressure; SBP, systolic blood pressure; DBP, diastolic blood pressure; MAP, mean arterial pressure; SNP, single nucleotide polymorphism*.

a *Recessive model. Statistically values are presented in bold*.

**Table 3:** Gene-based associations of *RNLS* with longitudinal BP changes and hypertension incidence over 14 years of follow-up.

**Gene**	**TPM**			
	**SBP change**	**DBP change**	**MAP change**	**Hypertension incidence**
*RNLS*	0.059	1.48 × 10^−5^	0.0085	0.017

*TPM, truncated product method; DBP, diastolic blood pressure; SBP, systolic blood pressure; MAP, mean arterial pressure*.

*All models were adjusted for the fixed effects of age, sex, and BMI, and the random effect for familial correlations*.

Next, we examined the relationships of *RNLS* SNPs with 5-year (2004–2009), 8-year (2004–2012), and 14-year (2004–2018) incidences of hypertension, which are shown in Table 4. SNP rs2576178 was significantly associated with the incidence of hypertension during 5 years of follow-up. SNPs rs796945, rs1935582, and rs2576178 were significantly associated with hypertension incidence over 14 years. Furthermore, *RNLS* was found to be significantly associated with hypertension incidence from gene-based analysis over 14-year follow-up (*P*_TPM_ = 0.017, Table 3) after adjustment for multiple testing.

**Table 4:** Association of individual SNPs with hypertension incidence.

**SNP**	**Incident hypertension (2004–2009)**		**Incident hypertension (2004–2012)**		**Incident hypertension (2004–2018)**	
	**Effect (95% CI)**	***P value***	**Effect (95% CI)**	***P value***	**Effect (95% CI)**	***P value***
rs10509540	−0.25 (−0.92 to 0.36)	0.428	−0.21 (−0.75 to 0.31)	0.435	−0.21 (−0.52 to 0.08)	0.157
rs7922058	−0.27 (−0.94 to 0.34)	0.397	−0.04 (−0.56 to 0.47)	0.871	−0.14 (−0.43 to 0.15)	0.348
rs999951	−0.22 (−0.89 to 0.40)	0.487	−0.16 (−0.70 to 0.36)	0.545	−0.22 (−0.52 to 0.08)	0.155
rs10887800	0.15 (−0.38 to 0.70)	0.589	0.10 (−0.35 to 0.54)	0.668	0.15 (−0.09 to 0.40)	0.217
rs796945	−0.29 (−0.82 to 0.21)	0.269	−0.22 (−0.64 to 0.20)	0.304	−0.24 (−0.48 to −0.01)	**0.042**
rs1935582	−0.23 (−0.76 to 0.30)	0.391	−0.30 (−0.75 to 0.14)	0.182	−0.29 (−0.54 to −0.04)	**0.023**
rs7076491	−0.35 (−1.18 to 0.38)	0.374	−0.23 (−0.86 to 0.37)	0.458	−0.20 (−0.54 to 0.13)	0.242
rs2296545	0.51 (−0.00 to 1.08)	0.060	0.11 (−0.32 to 0.55)	0.610	0.18 (−0.06 to 0.42)	0.138
rs2576178	−0.53 (−1.06 to −0.05)	**0.035**	−0.18 (−0.60 to 0.22)	0.378	−0.24 (−0.46 to −0.01)	**0.043**
rs17109290	−0.65 (−1.63 to 0.16)	0.147	−0.25 (−0.91 to 0.36)	0.428	−0.22 (−0.57 to 0.11)	0.197

*All models were adjusted for the fixed effects of age, sex, and BMI, and the random effect for familial correlations. SNP, single nucleotide polymorphism; CI, confidence interval. Statistically values are presented in bold*.

### Associations of Serum Renalase With BP Levels and the Risk of Hypertension in the Cross-Sectional Cohort Study

Participants with hypertension were older, and had higher prevalence of men, alcohol drinking, smoking, diabetes, family history of hypertension, and higher levels of BMI, heart rate, serum uric acid, fasting glucose, total cholesterol, triglycerides, LDL, serum creatinine, urinary albumin/creatinine, and lower levels of eGFR and HDL compared with those with normotension (Table 5).

**Table 5:** Characteristics of participants categorized by BP status in the cross-sectional cohort study (*n* = 2,392).

**Characteristics**	**All**	**Normotensive**	**Hypertensive**	***P*-value**
No. of subjects	2,392	1,904	488	–
Age (years)	43.0 (40.0–45.0)	43.0 (40.0–45.0)	43.0 (41.0–45.0)	0.001
Gender (M/F)	1,309/1,083	954/950	355/133	<0.001
BMI (kg/m^2^)	23.8 (21.9–26.0)	23.3 (21.5–25.4)	25.7 (23.7–27.7)	<0.001
SBP (mmHg)	123.1 ± 0.33	117.5 ± 0.23	144.8 ± 0.74	<0.001
DBP (mmHg)	77.0 ± 0.23	73.2 ± 0.18	91.8 ± 0.48	<0.001
Heart rate (beats/min)	73.0 (67.0–80.0)	73.0 (66.0–79.0)	75.0 (69.0–83.0)	<0.001
Alcohol consumption (*n*, %)	693 (29.0)	497 (26.1)	196 (40.2)	<0.001
Current smoking (*n*, %)	1,017 (42.5)	736 (38.7)	281 (57.6)	<0.001
Diabetes mellitus (*n*, %)	100 (4.2)	67 (3.5)	33 (6.8)	0.002
FH of hypertension (*n*, %)	1,249 (52.2)	923 (48.5)	326 (66.8)	<0.001
**Education level (** ***n*** **, %)**				0.359
Primary school or less	134 (5.6)	110 (5.8)	24 (4.9)	
Middle school	1,510 (63.2)	1,196 (62.9)	314 (64.3)	
High school	528 (22.1)	419 (22.0)	109 (22.3)	
College or more	218 (9.1)	117 (9.3)	41 (8.4)	
**Marital status (** ***n*** **, %)**				0.248
Married	2,271 (94.9)	1,810 (95.1)	461 (94.5)	
Divorced	88 (3.7)	72 (3.8)	16 (3.3)	
Unmarried or other	33 (1.3)	22 (1.1)	11 (2.2)	
**Level of physical activity (** ***n*** **, %)**				0.636
Almost no	979 (40.9)	781 (41.0)	198 (40.6)	
Light	1,257 (52.6)	997 (52.4)	260 (53.3)	
Moderate	99 (4.1)	77 (4.0)	22 (4.5)	
Heavy	57 (2.4)	49 (2.6)	8 (1.6)	
Serum uric acid (μmol/L)	277.9 (225.2–333.6)	269.7 (219.7–320.1)	321.2 (264.3–370.3)	<0.001
Fasting glucose (mmol/L)	4.57 (4.27–4.91)	4.53 (4.25–4.86)	4.72 (4.38–5.11)	<0.001
ALT (U/L)	19.0 (13.0–27.0)	18.0 (13.0–26.0)	23.0 (16.0–32.0)	<0.001
AST (U/L)	16.0 (13.0–20.8)	16.0 (13.0–20.0)	18.0 (14.0–23.0)	<0.001
Total cholesterol (mmol/L)	4.52 (4.05–5.01)	4.50 (4.02–4.97)	4.61 (4.19–5.15)	<0.001
Triglycerides (mmol/L)	1.34 (0.95–1.94)	1.26 (0.92–1.81)	1.65(1.18–2.32)	<0.001
LDL (mmol/L)	2.50 (2.14–2.90)	2.49(2.11–2.86)	2.55 (2.24–3.00)	<0.001
HDL (mmol/L)	1.15 (0.99–1.34)	1.17 (1.00–1.36)	1.08 (0.96–1.24)	<0.001
Serum creatinine (μmol/L)	76.6 ± 0.31	75.5 ± 0.32	81.2 ± 0.85	<0.001
eGFR (mL/min/1.73 m^2^)	78.2 (67.9–90.2)	79.8 (68.9–91.5)	72.5 (65.2–83.2)	<0.001
Urine albumin/creatinine (mg/g)	8.71 (5.64–15.25)	8.05 (5.30–13.15)	13.66 (7.76–30.42)	<0.001

*BP, blood pressure; BMI, body mass index; SBP, systolic blood pressure; DBP, diastolic blood pressure; FH, Family history; ALT, alanine aminotransferase; AST, aspartate transaminase; LDL, low-density lipoprotein; HDL, high-density lipoprotein; eGFR, estimated glomerular filtration rate; Non-normally distributed variables are expressed as the median (interquartile range). All other values are expressed as mean ± SE or n, %*.

**Figure 1:**
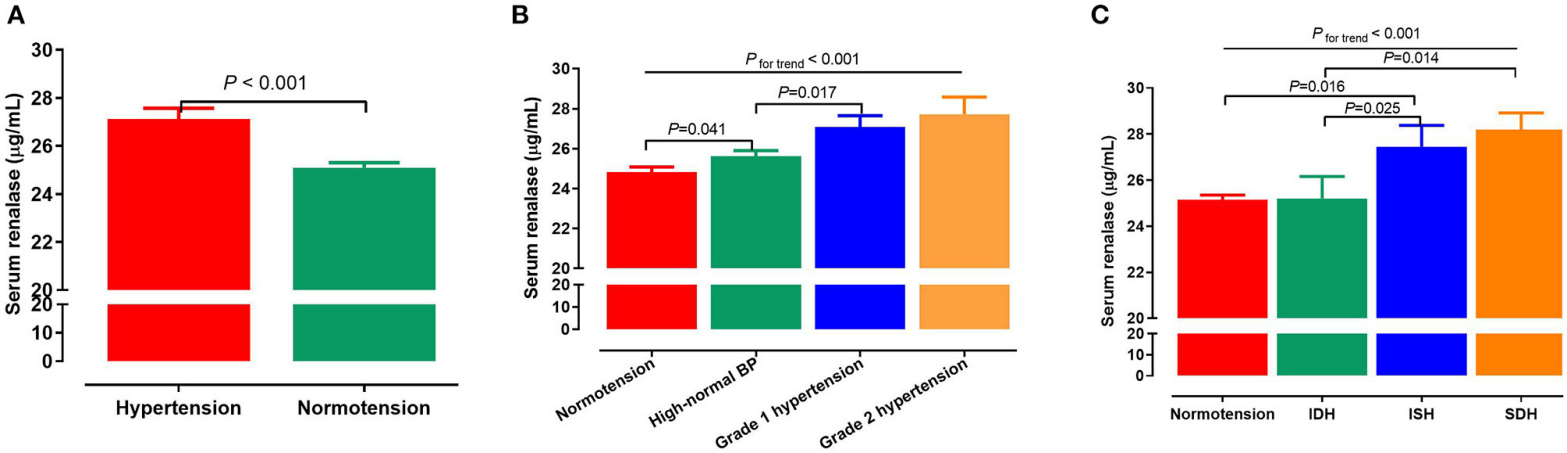
Associations of serum renalase with BP levels and hypertension. **(A)** Serum renalase levels in subjects with normotension and hypertension. **(B)** Serum renalase levels in subjects with different grades of BP, including normotension, high-normal, grade 1 and grade 2 hypertension. **(C)** Serum renalase levels in subjects with normotension and different hypertensive subtypes, including ISH, IDH, SDH. The differences between two groups were analyzed by Student's *t*-test, and analysis of variance (ANOVA) was used to test the linearity across different grades and subtypes of hypertension. BP, blood pressure; ISH, isolated systolic hypertension; IDH, isolated diastolic hypertension; SDH, systolic diastolic hypertension.

Serum renalase levels were higher in hypertensive subjects when compared with normotensive subjects (27.2 ± 0.4 vs. 25.1 ± 0.2 μg/mL, *P* < 0.001; Figure 1A). Furthermore, there was a stepwise increase in renalase levels from normotension, to high-normal, to grade 1 hypertension, and finally to grade 2 hypertension (24.8, 25.6, 27.1, and 27.7 μg/mL respectively, *P*_fortrend_ < 0.001; Figure 1B). We further examine serum renalase in different groups with normotensive and hypertensive subtypes. Participants with ISH had higher serum renalase levels than IDH group (27.4 ± 0.9 vs. 25.2 ± 1.0 μg/mL, *P* = 0.025]. Participants with SDH had higher serum renalase compared with IDH participants (28.2 ± 0.7 vs. 25.2 ± 0.9 μg/mL, *P* = 0.014; Figure 1C). Serum renalase was 25.2, 27.4, 25.2, and 28.2 μg/mL for subjects with normotension, ISH, IDH, and SDH, respectively (*P*_fortrend_ < 0.001).

**Figure 2:**
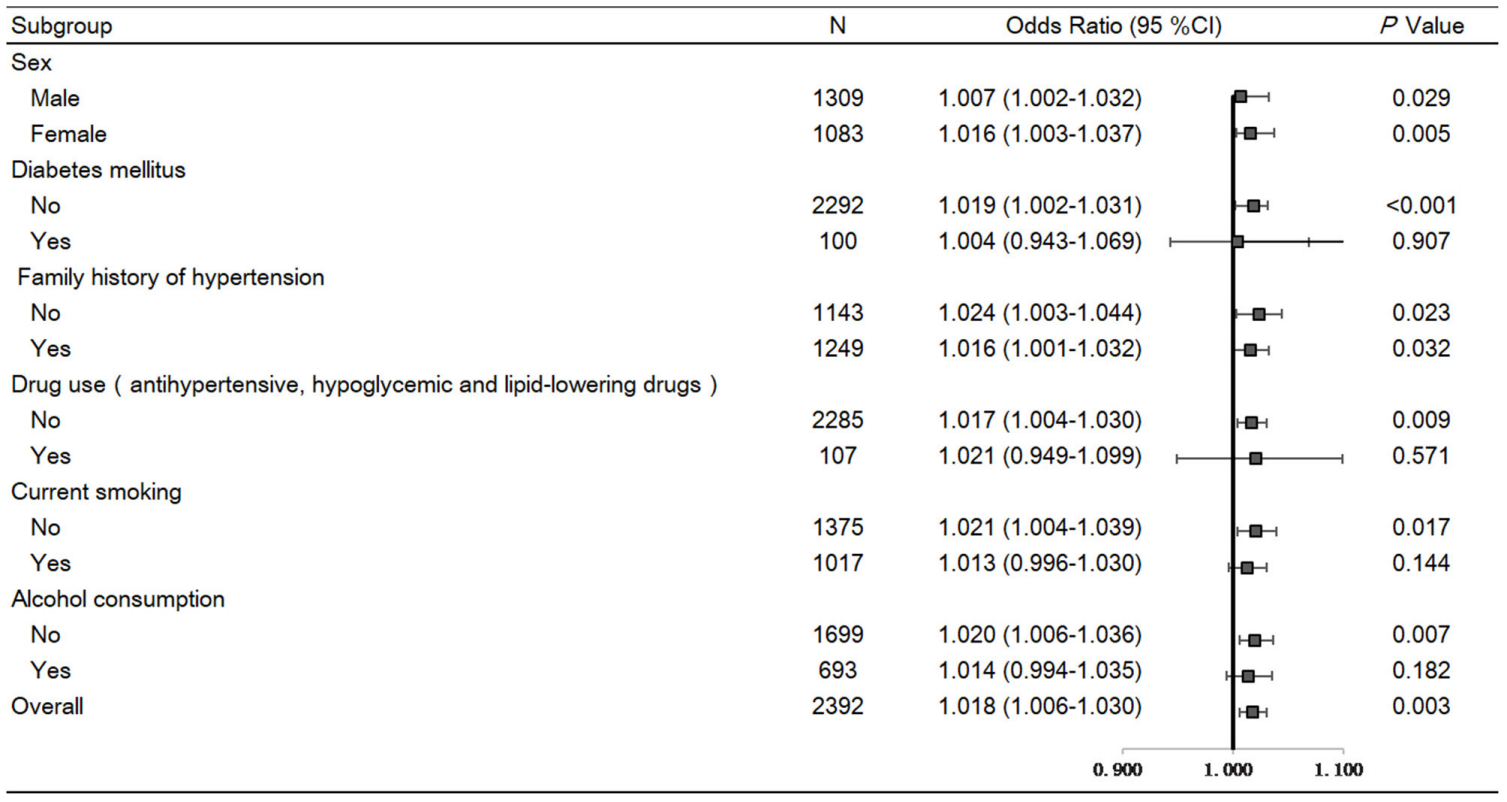
Forest plots of odds ratios (ORs) for serum renalase and risk of hypertension in a total of 2,392 subjects stratified by sex, BMI, diabetes, family history of hypertension, and drug use. Logistic regression analyses to test associations of serum renalase with hypertension risk after adjustment for age, smoke, BMI, eGFR, total cholesterol, triglycerides, fasting glucose. Values are the OR (95% confidence interval [95% CI]).

SBP levels were positively correlated with serum renalase (β = 0.056, *P* = 0.003), age, BMI, total cholesterol, eGFR, but negatively correlated with sex. In addition, DBP was positively correlated with serum renalase (β = 0.046, *P* = 0.015), age, BMI, total cholesterol, triglycerides, and negatively correlated with sex in all subjects (Supplementary Table 2). Furthermore, serum renalase [*OR* = 1.018 (1.006–1.030), *P* = 0.003] was significantly associated with the risk of hypertension after adjusting for multiple confounders (Figure 2).

Several sensitivity analyses were performed. First, removing subjects with diabetes or those taking antihypertensive, hypoglycemic and lipid-lowering drugs produced similar results (Figure 2). In addition, stratifying all participants by sex, BMI, family history of hypertension, did not change the results (Figure 2).

### Expression of Renalase in Human Renal Tissue in the Case-Control Study

Patients with hypertension were older, and higher levels of fasting glucose, total cholesterol, LDL, uACR, and lower levels of HDL compared with those with normotension. Serum creatinine, blood urea nitrogen and eGFR were similar between two groups (Table 6).

**Table 6:** Clinical characteristics of patients categorized by BP status in the case-control study (*n* = 193).

**Characteristics**	**All**	**Normotensive**	**Hypertensive**	***P*-value**
No. of subjects	192	55	137	–
Gender (M/F)	94/98	27/28	67/70	0.981
Age (years)	41.6 ± 1.2	35.3 ± 2.5	43.3 ± 1.2	0.002
BMI (kg/m^2^)	26.9 ± 2.3	31.9 ± 8.2	25.0 ± 0.4	0.404
Heart rate (beats/min)	87.6 ± 1.0	88.6 ± 1.7	87.1 ± 1.2	0.514
SBP (mmHg)	123.9 ± 1.4	110.3 ± 1.8	129.4 ± 1.7	<0.001
DBP (mmHg)	81.0 (74.0–88.0)	75.0 (67.0–79.0)	84.0 (76.5–92.5)	<0.001
Fasting glucose (μmol/L)	4.4 ± 0.1	4.3 ± 0.2	4.7 ± 0.9	0.473
Random blood glucose (μmol/L)	5.9 ± 0.2	5.3 ± 0.2	6.1 ± 0.2	0.029
Glycosylated hemoglobin (%)	5.7 ± 0.2	5.5 ± 0.1	5.8 ± 0.2	0.436
AST (U/L)	24.0 ± 0.9	22.6 ± 1.2	24.6 ± 1.2	0.334
ALT (U/L)	25.5 ± 1.6	21.8 ± 2.0	27.0 ± 2.1	0.147
Total cholesterol (mmol/L)	5.8 ± 0.2	5.5 ± 0.1	6.4 ± 0.4	0.034
Triglycerides (mmol/L)	2.2 ± 0.1	2.3 ± 0.3	2.2 ± 0.1	0.693
HDL (mmol/L)	1.3 ± 0.04	1.5 ± 0.1	1.3 ± 0.04	0.012
LDL (mmol/L)	3.8 ± 0.1	3.5 ± 0.1	4.3 ± 0.4	0.045
Serum sodium (mmol/L)	138.7 ± 0.8	138.8 ± 0.6	138.7 ± 1.0	0.931
Serum phosphate (mmol/L)	3.8 ± 0.03	4.0 ± 0.1	3.8 ± 0.03	0.005
Blood urea nitrogen (μmol/L)	5.2 ± 0.1	5.1 ± 0.2	5.3 ± 0.2	0.446
Serum creatinine (μmol/L)	54.0 ± 1.0	53.7 ± 1.8	54.1 ± 1.1	0.846
eGFR (mL/min/1.73 m^2^)	144.2 ± 3.0	152.5 ± 6.5	140.9 ± 3.2	0.114
Serum uric acid (μmol/L)	325.9 ± 6.5	307.5 ± 11.4	333.3 ± 7.7	0.071
C-reactive protein (mg/L)	2.0 ± 0.2	2.2 ± 0.4	1.9 ± 0.2	0.462
uACR (mg/g)	521.9 (239.1–1048.8)	332.9 (50.8–1018.2)	599.2 (257.2–1078.3)	0.024
**Pathologic diagnosis**				
Membranous nephropathy, *n* (%)	82 (42.7)	14 (25.0)	68 (49.6)	0.002
IgA nephropathy, *n* (%)	35 (18.2)	10 (17.9)	25 (18.2)	0.991
Mesangial and proliferative nephritis, *n* (%)	34 (17.7)	21 (37.5)	13 (9.5)	0.000
Systemic lupus erythematosus, *n* (%)	20 (10.4)	3 (5.4)	17 (12.4)	0.196
Purpura nephritis, *n* (%)	9 (4.7)	4 (7.1)	5 (3.6)	0.450
Focal segmental glomerulosclerosis, *n* (%)	4 (2.1)	1 (1.8)	3 (2.2)	0.871
Others, *n* (%)	8 (4.2)	2 (3.6)	6 (4.4)	0.816

*BP, blood pressure; BMI, body mass index; SBP, systolic blood pressure; DBP, diastolic blood pressure; ALT, alanine aminotransferase; AST, aspartate transaminase; HDL, high-density lipoprotein; LDL, low-density lipoprotein; eGFR, estimated glomerular filtration rate; uACR, Urinary albumin-creatinine ratio; Non-normally distributed variables are expressed as the median (interquartile range)*.

*All other values are expressed as mean ± SE or n, %*.

**Figure 3:**
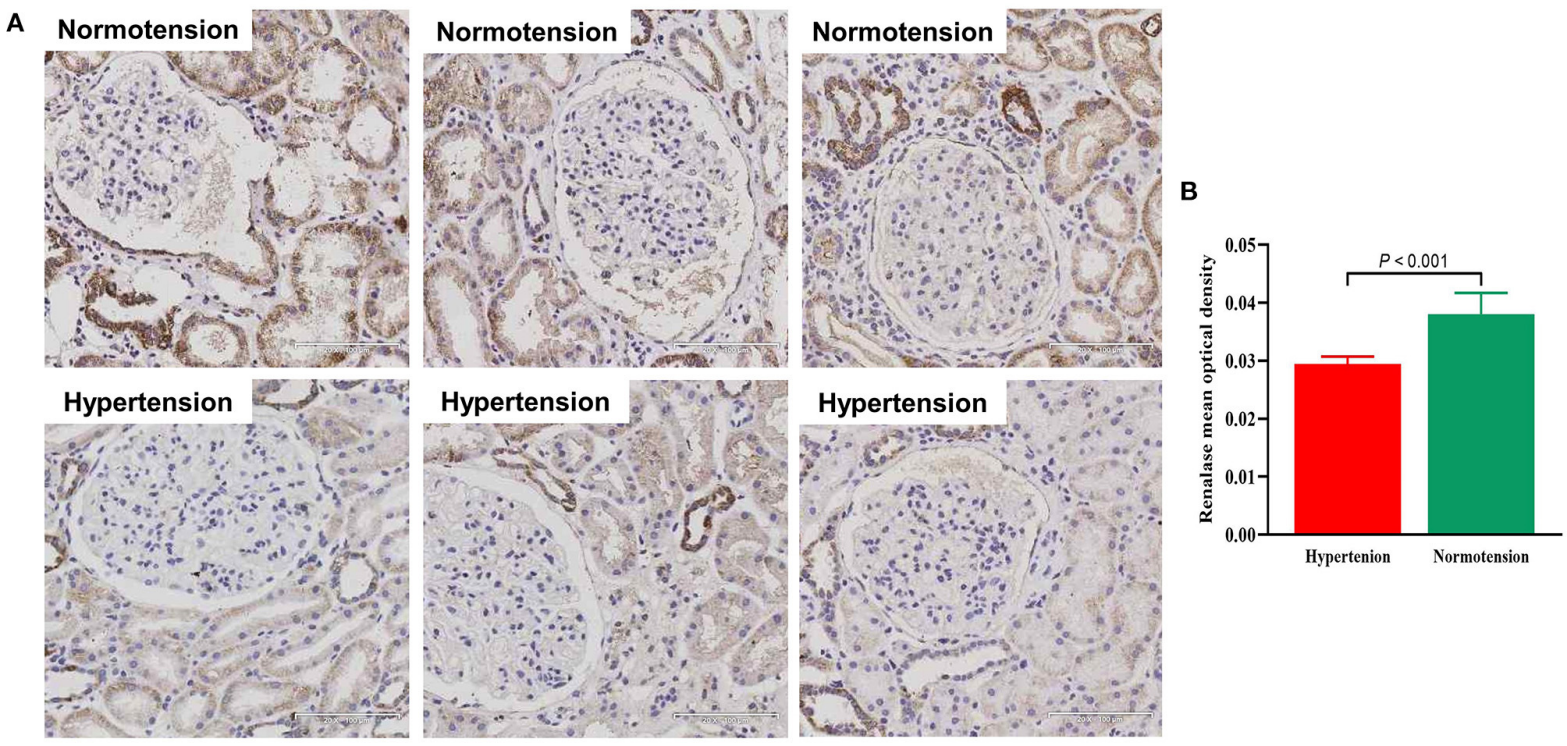
**(A)** Representative photomicrographs of renalase expression in human renal biopsy visualized by immunochemistry staining in normotensive group and hypertensive group (x200). **(B)** Bar graph showing quantitative analysis of renalase. Student's *t*-test was used to determine statistical differences between two groups.

We assessed the renalase expression levels and its locations in human renal biopsy by IHC. Renalase was strongly expressed in the renal tubules, particularly distal tubules, and scattered in the glomeruli of the normotensive patients (Figure 3A). In the hypertensive renal tissue, the number and intensity of renalase positive-stained area significantly decreased (Figure 3A). Quantification of renalase positive areas in the kidneys, as summarized in Figure 3B, indicated hypertensive group had lower expression of renalase as compared to the normotension group (0.030 ± 0.001 vs. 0.038 ± 0.004, *P* < 0.001).

## Discussion

To the best of our knowledge, this is the first study to investigate the associations of *RNLS* gene with BP levels, longitudinal BP changes and hypertension incidence in a Chinese cohort. We identified several novel *RNLS* SNPs that were significantly associated with BP levels, longitudinal BP changes and hypertension incidence after correcting for multiple testing. The gene-based analysis revealed that *RNLS* gene was significantly associated with longitudinal BP changes and hypertension incidence over time. These findings underscore potentially important contributions of renalase gene to long-term BP regulation. Moreover, it enhances our understanding of the genetic architecture of BP progression and hypertension.

The association between genetic variants of *RNLS* gene and hypertension was observed in several cross-sectional studies. For example, Zhao et al. (15) enrolled 2,586 Chinese subjects (1,317 with essential hypertension and 1,269 healthy controls), and showed a higher incidence of hypertension in subjects with G-allele rs2576178. Stec et al. (17) observed that the C-allele of rs2576178 was associated with a higher hypertension in hemodialyzed patients. However, the previous studies were cross-sectional, and the findings were inconsistent. Abdallah et al. (19) revealed that the frequencies of rs2576178 genotypes and alleles were not significantly different between normotensive and hypertensive subjects. In the present study, we identified 3 *RNLS* variants (rs2576178, rs796945, and rs1935582) that were significantly associated with incident hypertension over 14 years of follow-up. A significant gene-based association of *RNLS* and incident hypertension was also identified. SNP rs2576178 is located at the 5' flanking regions, and this polymorphism may influence the initiation of transcription or differential splicing (15). SNPs rs796945 and rs1935582 are located at the intronic region and have no inferred functional implication, based on the analysis by Fast SNP. However, evidence has suggested that intronic polymorphisms may be etiologically involved in the development of complex disorders (36). However, we cannot exclude that other SNPs in the same gene in variable grade of linkage disequilibrium with our SNPs could be implicated in BP/hypertension incidence. Future functional studies are needed to elucidate how the identified risk loci contribute to hypertension at the molecular and cellular level.

Renalase, mainly synthesized in the kidney and heart, is secreted into the blood (4, 6, 37). Several studies examining the relationship between circulating renalase and hypertension have yielded discordant results (Supplementary Table 3). For instance, hypertensive subjects showed significantly lower levels of serum renalase than their normotensive counterparts in 34 hemodialyzed patients (14) and 50 patients after surgical repair of aortic coarctation (12). Schlaich et al. (10) demonstrated that serum renalase was lower in 22 patients with resistant hypertension than in 4 normotensive controls. By contrast, some studies have shown opposed observation. Maciorkowska et al. (8) serum renalase was significantly higher in patients with hypertension (*n* = 121). These data were confirmed by Lemiesz et al. in 88 adolescents (9). In our previously established cohort of 2,392 subjects, we found that serum renalase were significantly higher in hypertensive subjects when compared with the normotensive ones. In this study, serum renalase was significantly associated with the risk of hypertension. The discrepant results of these studies may be attributed to their different renalase measurements in blood. Most studies estimated renalase levels with the use of commercially available enzyme-linked immunosorbent assay (ELISA) (8, 9, 11, 12, 14, 38, 39), whereas few studies used western blots (3, 10). The different study populations, sample sizes, and racial differences among these various studies may be other causes of the discrepant results (Supplementary Table 3).

In contrast to previous studies that only focused on dichotomous definitions of hypertension, our study reports stage-specific hypertension pursuant to the ISH global hypertension practice, including high-normal, grade 1 and grade 2 hypertension. To the best of our knowledge, this is the first study to quantify multi-state levels in the light of these criteria. We found that serum renalase showed a linear increase from normotension to grade 2 hypertension, indicating that circulating renalase may be a novel marker for identifying stages of hypertension. In addition, our study is the first to explore serum renalase levels in different hypertension subtypes. ISH was reported to be associated with an increased incidence of cardiovascular complications. IDH is associated with a low risk of cardiovascular disease mortality, while SDH carries a similarly high risk to ISH (40). Studies have established elevated SBP as a more reliable predictor for adverse cardiovascular outcomes compared to elevated DBP (41, 42). In the present study, we observed serum renalase levels in ISH group were significantly higher when compared with the IDH group and the normotension group. The serum renalase levels in the SDH group were higher than in the IDH group, but were similar to the ISH group. These data indicate that SBP is more closely related to serum renalase levels than DBP.

Renalase is extensively expressed in the kidneys, heart, intestines, liver, skeletal muscles, blood vessels, and central nervous system (4, 6). In the kidneys, renalase is widely expressed in renal tubule epithelial cells, glomeruli, mesangial cells, and podocytes (37, 43). However, few studies have explored the expression level of renalase in human kidney specimens. Huang et al. (44) reported that renalase expression detected by IHC was significantly lower in renal biopsy specimens with kidney disease than in normal kidney tissues, and it inversely correlated with renal injury and apoptosis. To our best knowledge, this is the first study aimed to compare the expression of renalase in human renal tissue from hypertension and normotension. Our results suggested that renalase was strongly expressed in the renal tubules, particularly distal tubules, and scattered in the glomeruli, which is consistent with the study of Wang et al. who showed that renalase was wildly expressed in kidney, and was secreted by tubule epithelial cells primarily (43). However, the previous study showed different renalase expression pattern (3), and different methods of these studies may be causes of discrepant results. Further, the expression in tubular epithelial cells was significantly lower in hypertensive group than in the normotensive group. Previous studies suggested that renalase levels were greatly affected by decreased renal function (45, 46). Our result was convincing because we excluded patients with renal dysfunction and focused on human samples rather than animal tissue. Interestingly, we observed that hypertensive subjects have lower expression of renalase in human renal tissue, but has higher levels of renalase in circulation. The mechanisms underlying this phenomenon remains to be explored. One could speculate that the increase in serum renalase reflect a counter-regulatory response to the increased inflammation and oxidative stress observed in hypertensive subjects (47). Renalase is expressed in macrophages and downregulates the inflammatory response (48). It would be informative but a large task to determine the expression of renalase in different tissues under hypertension.

Although the molecular mechanisms linking renalase to hypertension are not fully understood, several hypotheses have been proposed. Firstly, recombinant renalase exerts powerful and rapid hypotensive effects in rodents; this effect was suggested to be mediated by the degradation of circulating catecholamines, which could decrease cardiac contractility and heart rate (3). Wang et al. (49) further showed that serum renalase levels were correlated with catecholamine levels and SBP in CKD patients. In addition, BP is regulated by inhibiting the activation of the renal dopamine (DA) system. Sizova et al. (50) showed that renalase regulated the dopamine-dependent natriuresis and phosphaturia in 5/6 nephrectomized rats. Another study reported that the DA D1-like receptor agonist fenoldopam increased renalase expression in renal proximal tubule cells from Wistar Kyoto (WKY) rats, which was mainly attributable to D5 receptor (51). Finally, renal sympathetic nerve activity may be involved in the regulation of BP. Jiang et al. (52) found that renal denervation lowered BP, and upregulated the plasma renalase and renalase expression in the kidneys. Future studies are need to investigate mechanisms underlying this association.

The current study has several strengths. It provides the first evidence for the associations of renalase with hypertension and BP levels in humans from three different perspectives: genetic variations, plasma levels, and renal expression. Furthermore, our family-based cohort study was the first to examine the relationship between renalase and hypertension incidence longitudinally. In addition, this cross-sectional cohort study is the largest to date to detect serum renalase levels, and comprehensively explore its relationships with different BP levels, hypertensive grades, and subtypes. However, this study also has its limitations. The novel findings in our study need to be replicated in other cohorts with different genetic background. Moreover, due to the limited number of genotyped SNPs in *RNLS* gene, less frequent genetic variants may have been left out of the current study. Future research will be needed to explore their associations with longitudinal BP phenotypes and hypertension incidence.

Based on single-marker and gene-based analyses, the present study provides evidence for a role of the *RNLS* gene in longitudinal BP phenotypes and hypertension incidence. In addition, we reported for the first time that serum renalase level was significantly associated with different BP levels, hypertension grades, and subtypes. We also showed that renalase expression in human renal tissue significantly decreased in hypertensive patients. Findings from the current study indicate that renalase may play an important role in BP progression and development of hypertension. This work provides a basis for potential prevention and a possible therapeutic target for hypertension in the future.

## Data Availability Statement

The raw data supporting the conclusions of this article will be made available by the authors, without undue reservation.

## Ethics Statement

The studies involving human participants were reviewed and approved by First Affiliated Hospital of Xi'an Jiaotong University. The patients/participants provided their written informed consent to participate in this study.

## Author Contributions

YW and J-JM: conceived and designed the experiments. J-JM and W-HL: responsible for subject recruitment. G-LH, CChu, X-YZ, M-FD, TZ, Y-YL, QM, K-KW, YS, DW, YY, HJ, Z-JN, XZ, LW, Z-YM, C-HL, JZ, KG, and H-XL: performed the experiments. G-LH, CChe, QZ, and YW: analyzed the data. YW and CChe: drafted the paper. W-HL, JC, GD, and J-JM: edited and revised manuscript. All authors have read, critically revised, and approved the final manuscript.

## Funding

This work was supported by the National Natural Science Foundation of China (No. 81600327) (YW) and (Nos. 82070437 and 81870319) (J-JM), Natural Science Basic Research Program of Shaanxi Province (2021JM-257 and 2021JM-588), Institutional Foundation of the First Affiliated Hospital of Xi'an Jiaotong University (Nos. 2019QN-06 and 2021ZXY-14) (YW), the Clinical Research Award of the First Affiliated Hospital of Xi'an Jiaotong University of China [Nos. XJTU1AF-CRF-2019-004 (J-JM) and XJTU1AF2021CRF-021 (YW)], Research Incubation Fund of Xi'an People's Hospital (No. FZ-61). Grants from the Major Chronic Non-communicable Disease Prevention and Control Research Key Project of the Ministry of Science and Technology of China (2017YFC1307604 and 2016YFC1300104).

## Conflict of Interest

GD is a named inventor on several issued patents related to the discovery and therapeutic use of renalase. Renalase is licensed to Bessor Pharma, and GD holds an equity position in Bessor and its subsidiary Personal Therapeutics. The remaining authors declare that the research was conducted in the absence of any commercial or financial relationships that could be construed as a potential conflict of interest.

## Publisher's Note

All claims expressed in this article are solely those of the authors and do not necessarily represent those of their affiliated organizations, or those of the publisher, the editors and the reviewers. Any product that may be evaluated in this article, or claim that may be made by its manufacturer, is not guaranteed or endorsed by the publisher.
