# Supplementary material for: Associations of Renalase With Blood Pressure and Hypertension in Chinese Adults
Source: Front Cardiovasc Med. 2022 Feb 24;9:800427. doi: 10.3389/fcvm.2022.800427 (PMC8907541; doi:10.3389/fcvm.2022.800427)

**
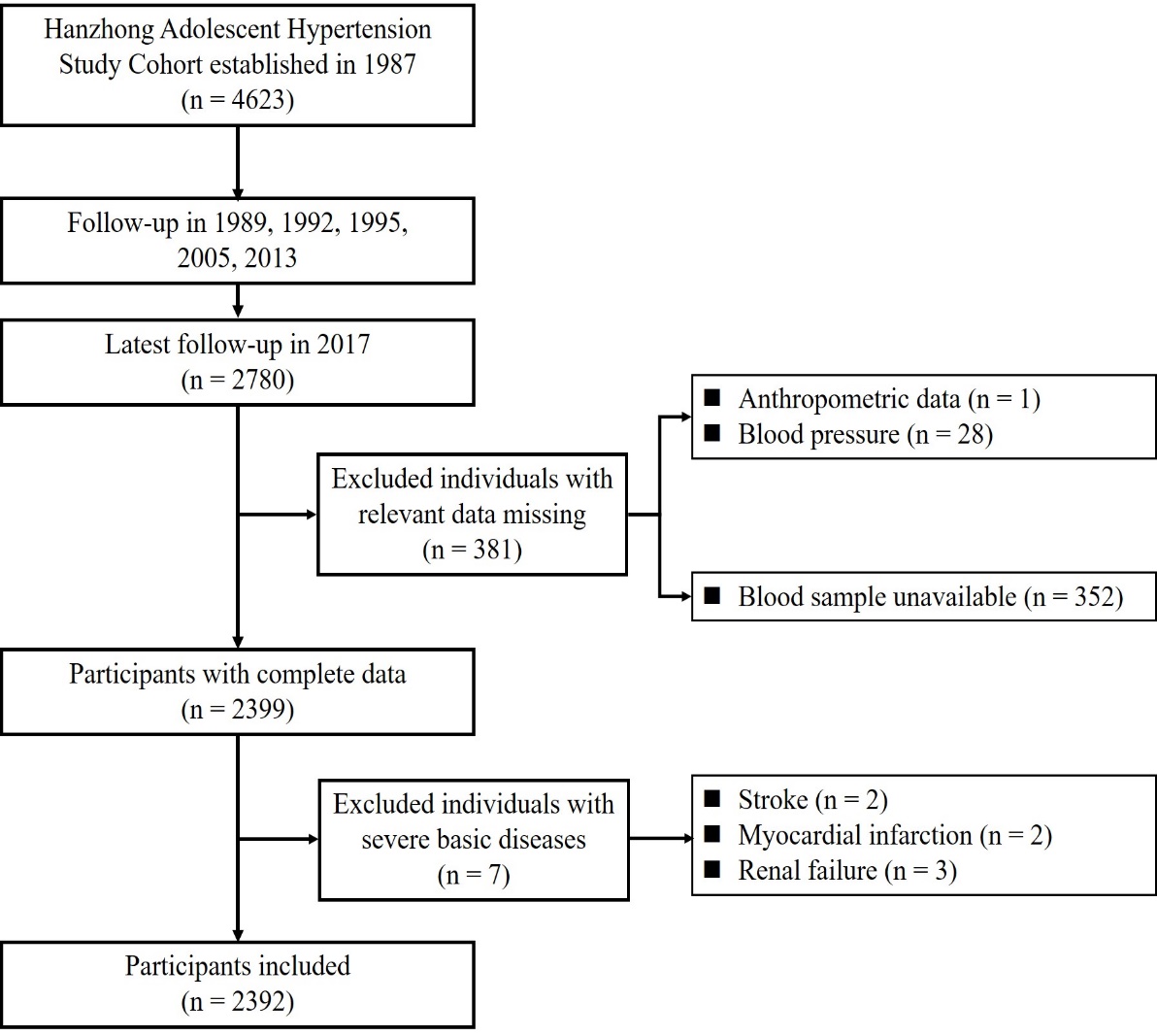
Figure S1.** Flow diagram for recruitment of participants in cross-sectional study.

**Table S1.** Information on genotyped SNPs of *RNLS*

| **SNP** | **Position** | **Region** | **Alleles *^a^*** | **MAF** | ***P*-value*^b^*^†^** | **Potential function prediction** |
| --- | --- | --- | --- | --- | --- | --- |
| rs10509540 | 88263276 | intron 7 | T/C | 0.250 | 0.376 | — |
| rs7922058 | 88274282 | intron 7 | A/G | 0.274 | 0.675 | miRNA |
| rs999951 | 88276610 | intron 6 | C/G | 0.240 | 0.250 | — |
| rs10887800 | 88316086 | intron 5 | A/G | 0.469 | 0.869 | — |
| rs796945 | 88391080 | intron 4 | T/C | 0.448 | 0.867 | — |
| rs1935582 | 88432773 | intron 4 | A/T | 0.483 | 0.620 | — |
| rs7076491 | 88471164 | intron 4 | T/C | 0.135 | 0.724 | — |
| rs2296545 | 88583080 | exon 1 | C/G | 0.403 | 0.226 | Splicing |
| rs2576178 | 88583641 | exon 1 | G/A | 0.451 | 0.401 | transcription factor binding site |
| rs17109290 | 88584404 | exon 1 | A/G | 0.129 | 1 | transcription factor binding site |

SNP, single nucleotide polymorphism; MAF, minor allele frequency. ^†^parents only (parental generation).

*^b^P* values of Hardy-Weinberg equilibrium test. *^a^* Alleles are presented as major: minor allele.

**Table S2.** Relationship between various characteristics and BP levels (n=2392).

| **Characteristics** | **Systolic Blood Pressure** | | **Diastolic Blood Pressure** | |
| --- | --- | --- | --- | --- |
|  | ***β*** | ***P* value** | ***β*** | ***P* value** |
| Serum renalase (μg/mL) | 0.056 | 0.003 | 0.046 | 0.015 |
| Gender (Male) | -0.192 | <0.001 | -0.258 | <0.001 |
| Age (years) | 0.124 | <0.001 | 0.070 | <0.001 |
| BMI (kg/m^2^) | 0.313 | <0.001 | 0.289 | <0.001 |
| Total cholesterol (mmol/L) | 0.049 | 0.011 | 0.052 | 0.007 |
| eGFR (mL/min/1.73m^2^) | 0.040 | 0.045 | 0.031 | 0.121 |
| Diabetes mellitus (n, %) | 0.028 | 0.140 | 0.023 | 0.229 |
| Triglycerides (mmol/L) | 0.030 | 0.141 | 0.049 | 0.016 |

BMI, body mass index; eGFR, estimated glomerular filtration rate.

**Table S3**. Characteristics of human studies on renalase and hypertension
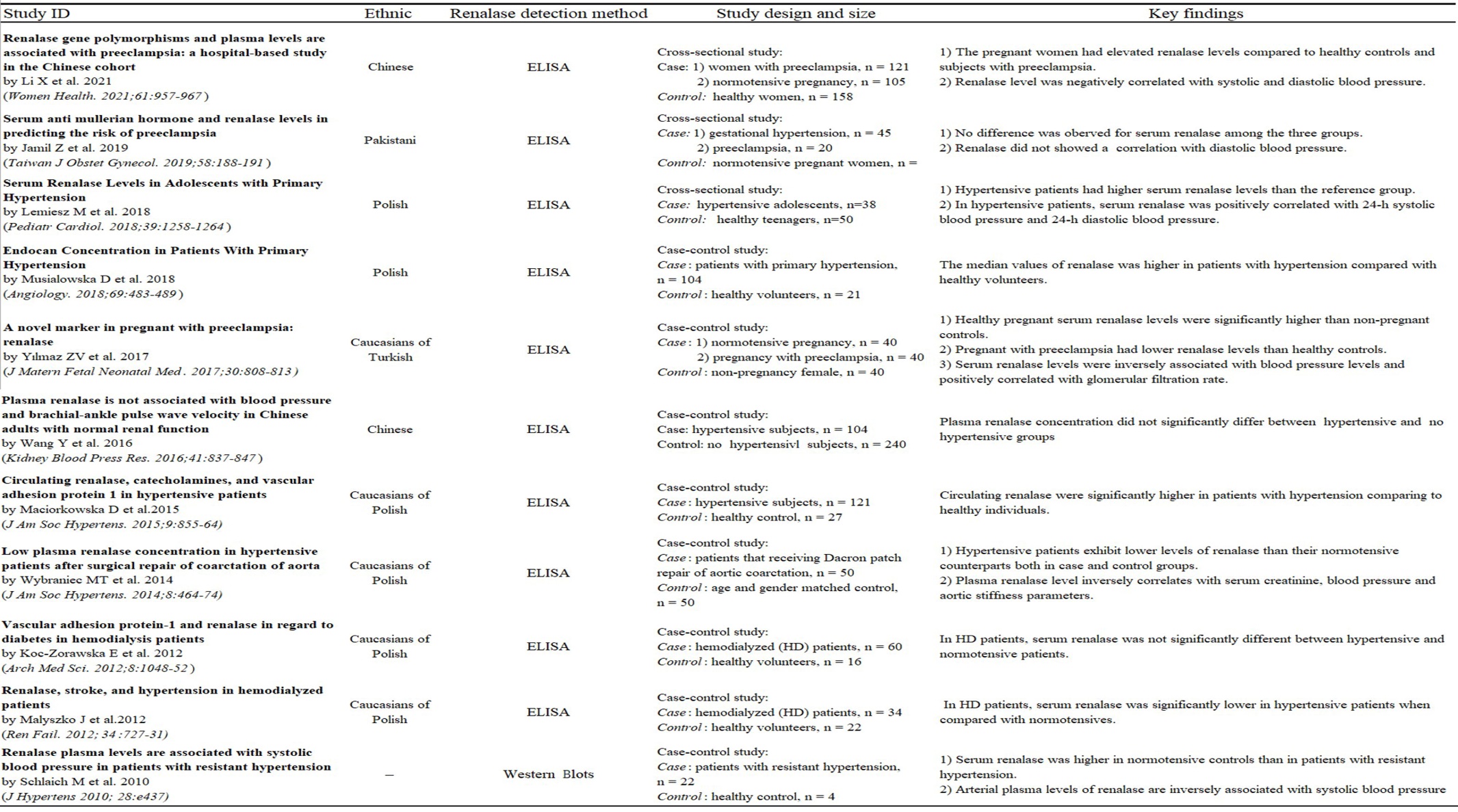

Supplement: Supplementary file 1 [file Data_Sheet_1.docx]
